# Gastrointestinal parasitic infestation in the Rock ptarmigan *Lagopus muta* in the French Alps and French Pyrenees based on long-term sampling (1987–2018)

**DOI:** 10.1017/S0031182020000517

**Published:** 2020-06

**Authors:** Angela Fanelli, Paolo Tizzani, Eric Belleau

**Affiliations:** 1 Department of Veterinary Sciences, University of Turin, Largo Paolo Braccini 2, 10090 – Grugliasco, Turin, Italy; 2 Groupement de Défense Sanitaire (GDS) des Alpes de Haute Provence, Barcelonnette, France

**Keywords:** France, *L*. *m*. *helvetica*, *L*. *m*. *pyrenaica*, parasite fauna, Rock ptarmigan

## Abstract

Data presented in this work represents the first record of parasites from the Alpine and Pyrenean *Lagopus muta* subspecies, providing valuable information to consider for conservation management. From 1987 to 2018, 207 Rock ptarmigans were collected in the framework of a long-term sanitary monitoring in France. Eight parasites were found in the Alpine Rock ptarmigan, and one in the Pyrenean subspecies. Only two parasites occurred with high prevalence in the Alpine Rock ptarmigan: *Capillaria caudinflata* (38.9%) and *Eimeria* sp. (34.7%). Prevalence of the other parasites (*Ascaridia compar*, Cestodes, *Amphimerus* sp. and *Trichostrongylus tenuis*) was lower than 20%. *Dispharynx nasuta* was found with a prevalence of 52.9% in the Pyrenean Rock ptarmigan. Overall, we found a spatially aggregated distribution of parasites in the northern French Alps, probably due to both favourable climatic conditions for parasite cycle and high host density. Statistical analyses indicated a positive effect of altitude and latitude on *C. caudinflata* occurrence whereas risk factors for *Eimeria* sp. were the distance from urban areas and land cover. In addition, the majority of the infested birds came from areas close to ski-pistes, where human disturbance increases the susceptibility to diseases, causing stress to wildlife.

## Introduction

The Rock ptarmigan *Lagopus muta* is a bird distributed with several subspecies in the arctic and alpine tundra of the Northern hemisphere. In contrast to the northern population, characterized by high density and occurring in wide and undisturbed habitats (Caizergues *et al*., 2003), the southernmost populations, which present a more fragmented distribution, have suffered a significant population reduction over the last decade. The decline may be explained by the climate change and human disturbance (Storch, 2000; Revermann *et al*., 2012; Imperio *et al*., 2013; Desmet, 2014; Novoa *et al*., 2014; Furrer *et al*., 2016), that are reducing even more the areas suitable for the presence of the species.

The Rock ptarmigan is classified as Least Concern (LC) by the International Union for the Conservation of Nature (IUCN, 2019), with no special conservation issues at the global level. However, National Red Data Books of several European countries, as well as the European Bird Directive (Council Directive 2009/147/EC) include it as a threatened species, pointing out the need for special conservation measures for its preservation (European Parliament and European Council, 2009).

Compared with other grouse species, scarce literature is currently available on the Rock ptarmigan (Zbinden and Hoerning, 1985), with most of the studies related to population dynamics, habitat and behaviour (Storch, 2000; Moss *et al*., 2010; Tizzani *et al*., 2020).

Specifically, few studies are available on the sanitary status of the species, and most of them have been carried out in the northern part of the species range, in Iceland (Skirnisson *et al*., 2012, 2016; Stenkewitz *et al*., 2016), Norway (Holmstad *et al*., 2005) and Japan (Murata *et al*., 2007; Matsubayashi *et al*., 2018). On the contrary, the health status of the Rock ptarmigan hasn't been fully investigated in Southern Europe where two subspecies are present at the border of the distribution range: *L. muta helvetica* and *L. muta pyrenaica*

To our knowledge, this is the first sanitary study carried out on the Pyrenean subspecies (*L. m. pyrenaica*). Moreover, it includes one of the few data currently published and available on the Alpine subspecies (*L. muta helvetica*), whose health evaluation is particularly difficult due to the fragmented distribution of the Alpine population, and to the difficulties to reach and sample the animals at high altitude areas. This is also one of the reasons why the studies on this species are usually limited to a very few number of samples.

Only Zbinden and Hoerning (1985), and more recently Fanelli *et al*. (2020), have carried out a study on the parasite community of wild Galliformes in the Alps, comparing the parasite diversity of the different host species. While the second study found no parasite affecting the Rock ptarmigan, the first found 13% of the birds infested by (in order of importance) *Capillaria*, *Coccidia*, *Hymenolepis*, *Heterakis* and *Trichostrongylus*.

Parasites on wild Galliformes have been proved by several studies to have a negative effect at the population level but at the same time their action can have a fine-tuned regulation. Specifically, this regulation could be expected mainly by parasite species showing longer co-evolutionary history with a host while other parasites, that met the host species more recently, could be expected to show an indiscriminate pathogenic effect (Hudson *et al*., 1992, 1998; Holmstad *et al*., 2005).

Moreover, species at the border of their distribution range are more sensitive to disturbing factors. As a consequence, the presence of parasites in a Rock ptarmigan population can be a real concern for the long-term conservation management.

Many abiotic environmental factors have an important role in the distribution, transmission and developmental success of parasites (Lafferty, 2009; Cable *et al*., 2017; Sanchis-Monsonís *et al*., 2019). Thus, understanding the factors that drive responses across parasite taxa in the Rock ptarmigan is essential to better tune conservation plans.

For these reasons, the purposes of this study are: (a) to increase the knowledge on parasites harboured by the southernmost populations of the Rock ptarmigan, both in terms of parasite community structure and distribution and (b) to assess the role of environmental factors on parasite infestation.

## Methods

### Study area and sample

Two *Lagopus* subspecies are reported in France: the Alpine Rock ptarmigan *L. muta helvetica* living in the Alps and the Pyrenean Rock ptarmigan *L. muta pyrenaica* whose range is limited to the Pyrenees. In our study, we collected samples from both subspecies. Most of the sampled animals were derived from birds killed during hunting activity (September–November), and few others found dead for other reasons (impact with cables, or predation). Usually, the whole carcases of the birds were collected and analysed. The sample was collected during the period 1987–2018.

The Alpine Rock ptarmigan is a small species of grouse Tetraonidae (Brenot *et al*., 2005) living in the higher subalpine and alpine zone between 1900 and 2600 m above sea level (Desmet, 1988). The Pyrenean Rock ptarmigan *L. m. pyrenaica* inhabits one of the most southernmost limits of the species range, around 400 km far from the Alpine population. Both subspecies are characterized by isolate and small populations, with low genetic variability.

**Fig. 1.**
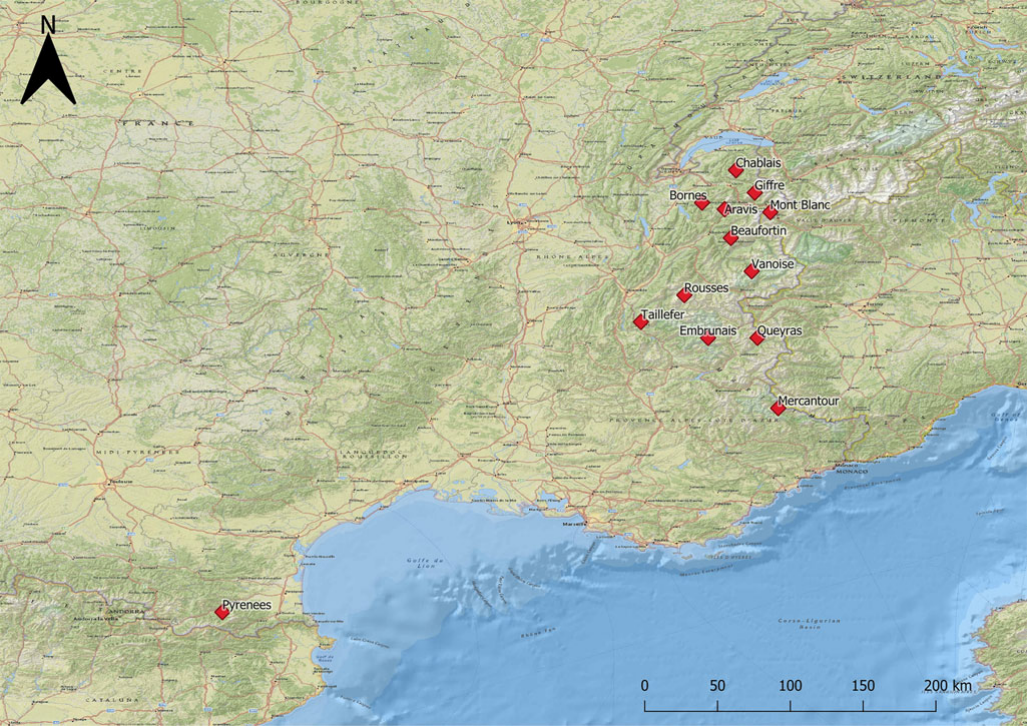
Study area: massifs sampled in the French Alps and French Pyrenees.

Samples coming from *L. m. pyrenaica* represented only 8.2% (17 out of 207 samples) of the animals analysed. All these animals originated from the Eastern Pyrenees Department, located in Southern France in the Occitanie region. The other 91.8% of the samples (190 out of 207 samples) were collected in the Alpine range from the *L. m. helvetica* subspecies. Twelve out of the 39 massifs comprising the French Alps and Prealps were investigated: four massifs belonging to the Prealps, low to medium altitude mountains contouring the western side of the Alps, and eight belonging to the Alps which are located in the Auvergne-Rhône-Alpes and Provence-Alpes-Côte d'Azur regions. Figure 1 shows the distribution of the sampled animals.

### Parasitological analysis

The gastrointestinal tract of the sampled animals was opened with a longitudinal incision, and the content of the individual sections (proventriculum, gizzard, small and large intestine) was analysed following the common parasitological standard techniques. Adult worms were then counted under a stereoscope (Maff, 1986).

To evaluate *Eimeria* sp. infestation, we examined fecal samples, collected from the rectum, using saturated sodium chloride flotation and formol ether sedimentation techniques. Eggs or oocysts were identified using a light microscope at ×40.

For *Amphimerus* sp., only visual inspection of liver presence of adult stage was carried out, thus only the presence of the parasite was noted, but not the quantity of parasites for each positive host.

Parasites were identified using a light microscope and the identification key suggested by Euzeby (1981, 1982). *Eimeria* sp. identification was done only at the genus level, as all the samples were frozen before the analysis, not allowing to evaluate the sporulate form of the parasite (needed for identification at the species level). Cestode identification was possible only for some positive animals, due to the reduced preservation status of some samples.

The same sampling and analytical methods were homogeneously applied through the whole period of study.

### Statistical analysis

All statistical analyses were carried out with R version 3.5.2 software (R Core Team, 2018). Epidemiological characteristics including prevalence (percentage of infected host individuals in each sample), intensity of infection (mean number of parasites per infected host) and abundance (mean number of parasites per host) were calculated for each parasite species. For *Eimeria* sp., the epidemiological indexes of intensity and abundance refer to the number of oocystis per gram of feces. Next, we used the package lme4 (Bates *et al*., 2015) to build a generalized linear mixed models (GLMMs) of the binomial family, with a logit link function to test whether environmental variables affect parasite occurrence. The environmental variables used in the model are listed in Table 1. The sampling area was considered as random term. Since the Pyrenean Rock ptarmigans had a very limited number of samples, animals from this area were excluded from the models. Moreover, because of the limited numbers of positive animals, it was possible to build a model only for *Capillaria caudinflata* and *Eimeria* sp., whereas only descriptive statistics was performed for the rest of the parasites. To avoid multicollinearity problem, we computed the variance inflation factors (VIFs) (Heiberger, 2018). Considering that none of the variable had a VIF >5, all of them were included into the GLMM. The best model was selected using a stepwise approach to minimized Akaike's information criterion (Akaike, 1973) whereas the likelihood ratio test was implemented to evaluate whether the response differs significantly by the random effect variable. In case no significant difference was detected for the random effect, a generalized linear model (GLM) was used. Finally, the area under the curve (AUC) value was computed to assess model performance.

**Table 1.** Environmental variables included in the model-building process

Variable	Source
Digital elevation model (DEM)	http://srtm.csi.cgiar.org/srtmdata/
Aspect [Northern (326–360; 0–45 degree), Eastern (46–135 degree), Southern (136–225 degree) and Western (226–325) exposure]	Derived from Digital Elevation Model (DEM) http://srtm.csi.cgiar.org/srtmdata/
Slope	Derived from Digital Elevation Model (DEM) http://srtm.csi.cgiar.org/srtmdata/
Longitude	Longitude of the sampled animals in UTM – WGS84 (32°N) coordinate
Latitude	Latitude of the sampled animals in UTM – WGS84 (32°N) coordinate
Distance from urban areas	Derived from CORINE Land Cover (CLC) https://land.copernicus.eu/
CORINE Land Cover (CLC) (sparsely vegetated areas, natural grasslands and bare rocks)	https://land.copernicus.eu/

## Results

### Gastrointestinal parasites

Eight parasites were found in the Alpine Rock ptarmigan, and one in the Pyrenean subspecies. Prevalence, mean abundance and intensity values, with confidence interval (CI) and standard deviation (s.d.) are presented in Table 2. All the values have been calculated at the host species level.

**Table 2.** Gastrointestinal parasite species from Rock ptarmigan *L. muta* in France

Parasite	Host species	Prevalence (CI_95%_)	Mean abundance (s.d.)	Mean intensity (s.d.)
*A. compar*	*L. m. helvetica*	1.6 (−0.2 to 3.4)	0.5 (5.4)	34.6 (31.3)
*C. caudinflata*	*L. m. helvetica*	38.9 (32.0–45.9)	8.8 (20.2)	22.5 (27.2)
Cestodes (including *Hymenolepis microps*, *Raillietina* sp., *Davainea tetraoensis*)	*L. m. helvetica*	14.2 (9.2–19.2)	4.1 (20.8)	28.5 (49.1)
*Eimeria* sp.	*L. m. helvetica*	34.7 (28.0–41.5)	229.8 (1267.3)	661.6 (2092.8)
*Amphimerus* sp.	*L. m. helvetica*	1.1 (−0.4 to 2.5)	NA	NA
*T. tenuis*	*L. m. helvetica*	5.3 (2.1–8.4)	0.9 (4.7)	16.5 (13.3)
*D. nasuta*	*L. muta pyrenaica*	52.9 (29.2–76.7)	NA	NA

Prevalence, 95% CI, mean abundance and mean intensity, standard deviation. For *Eimeria* sp. abundance and intensities values are referred to oocystis per gram of feces.

The results of the model are presented in Tables 3 and 4, for *C. caudinflata* and *Eimeria* sp., respectively.

**Table 3.** Results from GLMM for *C. caudinflata*

Fixed effects		
Variable	OR (CI_95%_)	*P* value
Aspect: northern exposure	0.4 (0.07–2.2)	0.3
Aspect: western exposure	0.3 (0.03–1.22)	0.08
Aspect: southern exposure	0.5 (0.7–3.6)	0.5
Digital Elevation Model (DEM)	0.4 (0.2–0.8)	0.02
Latitude	2.3 (1.5–5.3)	0.05
Distance from urban areas	0.8 (0.3–1.6)	0.5
CORINE Land Cover (CLC): grasslands	1.14 (0.21–1.72)	0.8
CORINE Land Cover (CLC): sparsely vegetated area	0.6 (0.2–1.7)	0.3
Random effects		
Group	Variance	s.d.
Sampling area	1.35	1.16
	Likelihood ratio test (*P* value = 0.003)	

*P* value <0.05 is significant.

**Table 4.** Results from GLM for *Eimeria* sp.

Variable	OR (CI_95%_)	*P* value
Digital Elevation Model (DEM)	1.44 (0.9–2.2)	0.08
Slope	1.36 (0.9–1.9)	0.07
Distance from urban areas	1.45 (1.2–2)	0.03
CORINE Land Cover (CLC): natural grasslands	2.3 (1.2–5.8)	0.05
CORINE Land Cover (CLC): sparsely vegetated areas	0.3 (0.1–0.6)	0.003

*P* value <0.05 is significant.

For *C. caudinflata*, the likelihood ratio test for the random parameter is statistically significant (*P* value = 0.003). This implies that the differences between the sampling areas contribute meaningfully to the model. The only significant factors retained in the model is the altitude (*P* value = 0.02) whereas the latitude *P* value present a marginal significance (exactly equal to 0.05). Our results indicate that there is a higher risk of *C. caudinflata* infestation at lower altitude [odds ratio (OR): 0.4] and an increased risk for animals living at higher latitude (OR: 2.3). Moreover, the predictive accuracy of *C. caudinflata* model is very high (AUC = 0.85).

Regarding the risk factors analysis for *Eimeria* sp. infestation, we built a GLM since the likelihood ratio test for the random effect was not statistically significant (*P* value = 1).

In the case of *Eimeria* sp., there is a clear and statistically significant increase of infestation risk as the distance from urban areas increases (OR: 1.45). Compared to the bare rock (used as reference baseline for land cover categories), the risk of infestation is 2.3 times higher for birds living in natural grasslands whereas those living in sparsely vegetated areas are at lower risk of infestation (OR: 0.3).

With regards to the other parasites, descriptive analysis points out that *A. compar*, *Amphimerus* sp. and *Trichostrongylus tenuis* were located only in the northern part of the study area. Moreover, the ptarmigans positive to *A. compar* and *Amphimerus* sp. were also located at lower altitude compared to the uninfested birds (*A. compar* 2254 *vs* 2633 m a.s.l. respectively; *Amphimerus* sp.: 2248 *vs* 2633 m a.s.l.). All the positive ptarmigans (10) infested by *T. tenuis* were reported from Vanoise massif only, with bare rocks (8 samples out of 10) and southern exposition (8 samples out of 10) as potential factors influencing the risk of infestation.

With regards to Cestodes, no specific pattern was revealed by the descriptive analysis, suggesting that no particular risk factors are driving the parasite distribution and prevalence at the population level.

Specific environmental risk factors analysis was not carried out for *L. m. pyrenaica*, as no accurate location of the samples was available.

## Discussion

Our study represents a comprehensive survey on the gastroenteric parasites community of the Rock ptarmigan subspecies living in the French Alps (*L. muta helvetica*) and French Pyrenees (*L. muta pyrenaica*). To date, information regarding the prevalence and diversity of parasites in the southernmost range of the species has been very limited. This is also the first work investigating the parasites of Rock ptarmigan based on a relatively large sample, covering a wide part of the species range in France and characterized by a long-term sampling (1987–2018). In comparison with the one found in the work of Zbinden and Hoerning (1985), or usually reported for other Alpine Galliformes hosts, a relative rich parasite richness was reported in our study. Moreover, the occurrence and distribution of the parasite at the population level seems to be influenced, at least in some species, by environmental variables. Regarding this last point, it is important to highlight that the environmental factors identified, have to be considered cautiously as they relate to the location where birds were collected, without considering their capacity to move inside the individual home range.

The different host parasite richness detected can be explained by different reasons. Even if the difference could be due to an unbalanced sample size, with a lower number of birds sampled in the Pyrenees, the difference found in the two subspecies might also be due to the geographical locations of the samples. Indeed, all the Pyrenean Rock ptarmigans sampled in our study came from the Eastern Pyrenees which are characterized by very dry climatic conditions, comparable with the ones recorded in the Southern Alps that probably do not allow the proper development of parasite cycles. In the Pyrenees, the amount of precipitation is greater in the western range. Most of the humidity coming from the Atlantic Ocean is dropped on the Western and Central Pyrenees, and the air arrives in the eastern part of the region mostly dry. This is also the reason why no glaciers are present in the Eastern Pyrenees (Hugh, 1911; Maris *et al*., 2009). A parasite richness is equal to 1 in the Pyrenean subspecies, due to a parasite species known for its pathogenic potential (*Dispharynx nasuta*). This is extremely important for several reasons: (i) while *D. nasuta* is reported in other avian species (Carreno, 2008), this is the first report of a parasite in this host species and in *Lagopus* genus in general, (ii) the parasite has been reported with quite high prevalence in our study and it seems to have an important pathogenic effect on the host (Rickard, 1985). For these reasons, the presence of *D. nasuta* could have very important effects on the population dynamics, and should be carefully monitored in the future. However, we cannot infer anything more because of the small sample size. Further studies with a larger sample size should be carried out in the future to better investigate the parasite community in *L. m. pyrenaica*. Indeed, our data are the first attempt to investigate the parasite community in this sub-species.

In the Alpine Rock ptarmigan, prevalence of the parasites varied, being *C. caudinflata* (37.5%) and *Eimeria* sp. (33.5%) the most common species detected. *A. compar*, *Amphimerus* sp., *T. tenuis* and *Cestoides* were present only in few samples. Indeed, these species are rare in *L. muta* whereas more common in other hosts belonging to the *Lagopus* genus (Dobson and Hudson, 1992; Dæhlen, 2003).

Compared with Fanelli *et al*. (2020), who have found the Rock ptarmigan living in the Italian Alps free of parasites, this study presents a parasite richness higher than the one detected by Zbdinden and Hoerning (1985) and similar, in terms of different parasite genus, to the one detected by Holmstad *et al*. (2005) in Norway and Skirnisson *et al*. (2012) in Iceland. However, a comparison with these studies is difficult, due to the different epidemiological situations of the ptarmigan populations, and due to the distinct population and environmental conditions. Making reference to the work of Fanelli *et al*. (2020), that investigated the wild Galliformes parasite community (in three host species: *L. m. helvetica*, *Tetrao tetrix tetrix* and *Alectoris graeca saxatilis*) in an area very close to the French Alps, it is interesting to notice that the parasite richness at the community level in this work is significantly lower, as well as the prevalence of *C. caudinflata* (detected in only 1.2–10% of the host species). At the contrary, *A. compar* in the work of Fanelli *et al*. (2020) was the most prevalent species. Also, parasite abundance and intensity described in our study were much higher than the one reported in Fanelli *et al*. (2020). These differences in parasite community richness and structure strongly support the need to carry out more specific study to better investigate the complex relationship among host, parasite and environment at the Alpine level. In the Alps, most of the Galliformes population exist in a situation of metapopulation, with possible significant differences among subpopulations, in terms of sanitary status and its possible impact on the long-term dynamics.

From a spatial point of view, we found an aggregated distribution of parasites in the northern part of the study area, probably due to the concomitant presence of favourable climatic conditions and high host density (census data from the Observatoire des Galliformes de Montagne – http://www.observatoire-galliformes-montagne.com/Lagopede-alpin.html).

In particular, altitude and latitude were significantly associated with *C. caudinflata* infestation. The majority of the infested ptarmigan came from the French Prealps which are characterized by lower elevation than the Alps, and are located in the northern part of the study area. The combination of these factors allows probably the parasite eggs to develop under more favourable climatic conditions. Indeed, significant climatic differences exist in the Alpine range, with the northern part receiving higher precipitations (Durand *et al*., 2009).

Several studies have evaluated the impact of environmental factors on the development, survival and distribution of parasites characterized by a cycle with free-living stages (Stromberg, 1997; O'Connor *et al*., 2006); however, very few studies have been carried out on wildlife for such long period of time (Iacopelli *et al*., 2020). Our finding is in line with the study of Fanelli *et al*. (2020) who found a higher prevalence of gastrointestinal parasites in wild Galliformes living at higher latitude. As in this study, these authors interpreted latitude as a proxy of the climatic and environmental conditions required for the development of free-living stages of the parasite.

Interesting insights are available also from the model for *Eimeria* sp., showing a higher risk of infestation with the increase of the distance from urban areas. The distance from urban areas acts in this case as a proxy for a different factor. From a visual exploration of the sampling points, it appears that most of the infested animals were close to the main ski-pistes that are located far away from urban zones in our study area. The distance from urban areas in our case should be considered as ‘close to urban-disturbed places like ski-pistes’. Indeed, it has been demonstrated that in the disturbed areas there is a strong correlation with coccidiosis (Giraudeau *et al*., 2014). In the Alps, the construction of ski-pistes has widely impacted the ecosystem, with a negative ‘edge effect’ on wild bird populations (Caprio *et al*., 2014). In addition to the direct impacts on fauna, flora and microbiota, the edge effect favours transmission of parasites to wild birds and susceptibility to parasites among wild birds (Oliveira *et al*., 2017). The negative effect of the presence of ski areas have been also demonstrated by Belleau (2013), in another study reporting a higher prevalence of *C. caudinflata* in Galliformes coming from human disturbed habitats.

In this way, a similar pattern can be seen also for *A. compar* and *Amphimerus* sp. distribution. In fact, despite the parasites infection affects few individuals, the positive samples are located in areas close to the ski-pistes. The anthropization can be from one side stressful to wild birds, as it leads to a chronic elevation of circulating glucocorticoids, impairing immunity in birds, and from the other side, increases the risk of higher presence of parasitic infectious stages (Bourgeon and Raclot, 2006; Giraudeau *et al*., 2014; Cable *et al*., 2017). In addition, the positive animals derived also from an *L. m. helvetica* population at low altitude. The combination of these factors has probably increased even more the risk of parasite occurrence.

In the *Eimeria* sp. model, a further result is related to the different risk of infestation according to the land cover, with higher risk in natural grasslands and lower in sparsely vegetated areas compared to bare rocks (that is used as baseline value for land cover risk evaluation). This result might be explained by the different amounts of soil moisture content. Indeed, land humidity might influence the oocyst survival and development, with moisture inducing higher sporulation ability (Nesheim *et al*., 1979; Venkateswara Rao *et al*., 2015).

Birds affected by *T. tenuis* came from a very specific area, showing a cluster in the Vanoise massif. This result is of particular interest, since the presence of this parasite has never been reported in France (Belleau, personal communication). Specifically, *T. tenuis* has been previously described in northern ptarmigan populations (Stenkewitz *et al*., 2016) and widely studied in other host-species (Shaw and Moss, 1989; Freehling and Moore, 1993; Hudson and Dobson, 1997). However, due to the limited number of positive samples, no further statistical analysis was performed to evaluate environmental risk factors associated with the infestation. The presence of this parasite only in the Vanoise area could be due to particularly favourable environmental conditions. However, it is important to notice that there was no further report of this parasite since 1992, following several years with very dry summers that probably caused the local extinction of the nematode.

Although only prevalence was estimated for Cestodes infestations, this study made an important contribution to the understanding of the parasite community of the Alpine *L. muta* subspecies, describing three new parasite species. Moreover, the prevalence is among the most fundamental measures in epidemiology and it may be used to compare disease burden across locations or time periods. Additionally, there have been only few field studies published on this subject, and none of them on the Rock ptarmigan (Avery, 1969; Dick and Burt, 1971, 1971; Wissler and Halvorsen, 1977; Delahay, 1999; Dæhlen, 2003).

To conclude, the broad implication of our study is to increase the information available on the structure and variability of the parasite community in the Rock ptarmigan. As parasites may impact on population dynamics and increase the risk of local extinction for threatened species (Herrera and Nunn, 2019), future studies should explore the sanitary status of this species to correctly plan conservation measures. In particular, this work highlighted as the parasite in Rock ptarmigan have a clustered distribution, so specific monitoring plan should be carried out to assess the local health status and propose specific measures for a better conservation management.
